# Community knowledge and perceptions of social, ecological, and behavioural risk factors for mpox in Burundi: A nationwide cross-sectional survey

**DOI:** 10.1371/journal.pgph.0007383

**Published:** 2026-10-27

**Authors:** Néhémie Nzoyikorera, Emmanuelline Mukama, Jonas Ngendakumana, Ferdinand Nsengimana, Roger Ciza, Marie Noelle Uwineza, Alain Tamba Yombouno, Duncan Kabiito Matovu, Rodgers R. Ayebare, Cathy Asiimwe Tabaro, Anatole Nkeshimana, Idrissa Diawara, Mosoka Papa Fallah, Joseph Nyandwi

**Affiliations:** 1 National Reference Laboratory, National Public Health Institute, Bujumbura, Burundi; 2 National Public Health Institute, Bujumbura, Burundi; 3 Africa Centres for Disease Control and Prevention, Bujumbura, Burundi; 4 Department of Global Health Security, Infectious Diseases Institute, College of Health Sciences, Makerere University, Kampala, Uganda; 5 Africa Centres for Disease Control and Prevention, Addis Ababa, Ethiopia; 6 Research Laboratory of Microbiology, Infectious Diseases, Allergology and Pathogen Surveillance (LARMIAS), Mohammed VI Faculty of Medicine, Mohammed VI University of Sciences and Health (UM6SS), Casablanca, Morocco; 7 Mohammed VI Higher Institute of Biosciences and Biotechnologies, Mohammed VI University of Sciences and Health (UM6SS), Casablanca, Morocco; 8 Mohammed VI Center for Research and Innovation (CM6RI), Rabat, Morocco; 9 Faculty of Medicine, University of Burundi, Bujumbura, Burundi; The University of Sydney, AUSTRALIA

## Abstract

Mpox remains an important public health concern in Africa, particularly following the spread of clade Ib in several countries. In Burundi, limited information is available on population knowledge, risk perception, preventive behaviours, and willingness to accept mpox vaccination. This study assessed social and behavioural factors associated with mpox preparedness among the general population. We conducted a nationwide cross-sectional survey between March and December 2025 across five provinces of Burundi. A total of 5,177 participants aged 15 years and older were interviewed using a structured questionnaire adapted from the Africa CDC behavioural and social sciences framework for mpox. Sampling weights were not applied; therefore, findings should be interpreted as estimates among surveyed participants rather than nationally weighted population estimates. Univariate analyses were conducted to describe key variables, while bivariate and multivariable analyses using modified Poisson regression models with generalized estimating equations were performed to identify factors associated with mpox knowledge and prevention and control practices. Overall, most participants had heard about mpox (94.7%). Many correctly identified skin rash (94.4%) and fever (84.3%) as common symptoms, and most recognized direct contact as a mode of transmission (86.6%). However, 21.4% of participants incorrectly identified bacteria as the causative agent and 24.5% did not know the causative agent. Radio was the main source of information (82.0%) and the most trusted source (87.0%). Higher income was associated with better mpox knowledge. Prevention and control practices varied by knowledge level, income, marital status, and HIV status. Although most participants (86.1%) were unaware of the availability of a vaccine, 76.5% reported willingness to accept vaccination if available. Despite high awareness and willingness to accept vaccination, important gaps in mpox knowledge remain, particularly among lower-income groups. Strengthening accessible and targeted risk communication strategies will be essential to improve mpox preparedness in Burundi.

## Introduction

Since the eradication of smallpox, mpox has become the most important Orthopoxvirus affecting humans and remains a growing public health concern [1,2]. Since May 2022, outbreaks have been reported well beyond the traditionally endemic regions of Central and West Africa, resulting in sustained transmission across multiple continents. By 2025, more than 170,000 laboratory-confirmed cases and over 460 deaths had been reported from more than 140 countries worldwide [3]. The epidemiology of the disease has also changed with the emergence of clade Ib, first identified in eastern Democratic Republic of the Congo (DRC) and subsequently reported in several African and non-African countries [4].

In response to the rapid spread of clade Ib, the Africa Centres for Disease Control and Prevention (Africa CDC) declared a Public Health Emergency of Continental Security (PHECS) on 13 August 2024 [5], followed by the World Health Organization (WHO) declaration of a Public Health Emergency of International Concern (PHEIC) a day later [6]. Although the PHEIC was lifted in September 2025, mpox continues to circulate in several countries, particularly in Africa, where community transmission remains a major public health challenge [3,7].

Burundi reported its first confirmed mpox cases on 23 July 2024 [8,9]. Since then, the country has recorded continued transmission and has implemented a range of response measures, including enhanced surveillance, case management, risk communication, and community engagement activities [10]. According to the Burundi National Mpox Surveillance Database maintained by the Ministry of Public Health, 4,727 laboratory-confirmed cases and one death had been reported by 7 May 2026.

Mpox is transmitted mainly through close contact with infected individuals, contaminated materials, or infectious lesions, including during sexual contact [11,12]. However, recent outbreaks have shown that disease transmission and control are influenced not only by biological factors but also by social and behavioral factors. Recent studies from African settings have demonstrated substantial variability in mpox knowledge, risk perception, and preventive behaviours, emphasizing the importance of context-specific risk communication and community engagement strategies [13]. People’s knowledge of the disease, perception of risk, trust in information sources, and ability to adopt preventive measures all play an important role in shaping outbreak dynamics. The socio-ecological model provides a useful framework for understanding how these factors operate at individual, interpersonal, community, and structural levels to influence health behaviors [13–15].

Despite the ongoing outbreak in Burundi, little is known about public knowledge, risk perception, preventive practices, trust in information sources, and acceptance of mpox vaccination in Burundi. Such information is needed to strengthen preparedness and guide risk communication and community engagement strategies. This study was designed to address the knowledge gap regarding factors associated with community knowledge, perceptions, and preventive practices related to mpox in Burundi, which are important for informing risk communication and community engagement strategies. Therefore, this study assessed mpox preparedness among the general population in Burundi by examining knowledge, risk perception, preventive practices, trusted information sources, and vaccine acceptance, and explored factors associated with knowledge and preventive behaviours.

## Methods

### Ethics statement

The ethical approval to conduct this study was given by the National Ethics Committee in Burundi (Reference number: CNE/02/2025). All participants for the survey provided individual written and signed consent for the interview. For participants between 15–17 years, parental or guardian consent was obtained in addition to assent from the participants.

### Study design and setting

This was a cross-sectional population-based survey conducted using a standardized questionnaire derived from the Africa CDC Mpox Behavioral and Social Sciences Survey protocol (S1 File). The survey was conducted in the five newly established provinces of Burundi (Bujumbura, Gitega, Buhumuza, Burunga, and Butanyerera), which together cover the entire national territory following the 2023 administrative reform. Therefore, the study was designed to achieve broad population-level geographic coverage across all provinces of Burundi, rather than a fully weighted nationally representative probability sample.

A multistage cluster-based sampling approach was used to achieve geographic coverage across the five provinces of Burundi included in the survey. The sampling process involved the selection of health districts, communities, households, and eligible participants. Within selected clusters, households and eligible participants were selected using predefined random selection procedures described in the Africa CDC survey protocol. Briefly, eligible households within selected communities were identified following field implementation procedures (S2 File), and one eligible participant was selected from each selected household according to the protocol. Sample allocation was guided by the survey protocol and operational feasibility to ensure coverage of the five provinces and different geographic contexts. The allocation was not designed to produce province-specific or nationally weighted estimates. Because sampling weights were not applied during analysis, the findings should be interpreted as describing the surveyed population and not as weighted national estimates. Data collection occurred from 29^th^ of March to 9^th^ April 2025 in Bujumbura, and from 18^th^ to 31^st^ December 2025 in the remaining provinces of Gitega, Buhumuza, Butanyerera and Burunga.

### Study population and recruitment

All participants in this study were recruited from selected communities within the study provinces. Eligible participants were individuals aged 15 years and above and who provided informed consent prior to participation. Recruitment was conducted within selected communities according to the Africa CDC survey protocol and field implementation procedures. Local community structures supported the identification and mobilization of eligible households and participants. Households selected for participation were not known to have active mpox cases at the time of recruitment, reducing potential bias due to acute illness clustering. The household contacts did not have signs of mpox at recruitment time.

### Sample size and sampling

The sample size was calculated using a formula for cross-sectional studies that considers both precision and statistical power [16]: n = ((Z_1_ ₋ α/2 + Z_1_ ₋ β)^2^ × [P(1 − P)]) / d^2^ where *n* is the required sample size, *Z*_1_ ₋ α/_2_ is the standard normal value for a 95% confidence level (1.96), *Z*_1_ ₋ β is the standard normal value for 90% power (1.28), *P* is the estimated prevalence of mpox knowledge (29%) based on a previous study from Nigeria [17], and *d* is the margin of error (5%). The calculated sample size was 865 participants. After adding 10% for possible non-response, the minimum sample size increased to 951.

The study followed the standardized Africa CDC protocol for assessing the socio-ecological and behavioral drivers of mpox in African Union Member States. Because the survey used a multistage cluster sampling approach, the sample size was adjusted using a design effect. According to the protocol, countries with more than 200 reported mpox cases were assigned a design effect of 5. At the time of protocol development, Burundi had reported more than 200 mpox cases; therefore, a design effect of 5 was applied, resulting in a target sample size of 4,755 participants.

A multistage cluster-based sampling approach was used to recruit participants from the five provinces included in the survey. The sampling process involved sequential selection of health districts, communities, households, and eligible participants within households. The five provinces were included to provide geographical coverage during the mpox outbreak response and to capture different socio-demographic contexts. Probability proportional-to-size (PPS) sampling was not applied because the survey was designed to describe mpox-related awareness, perceptions, knowledge, and preventive practices among the surveyed population rather than to generate population-weighted provincial or national estimates. Within selected households, individuals aged ≥15 years were eligible for participation according to the survey protocol. Because individual selection probabilities were not available, sampling weights were not applied during analysis. Therefore, the findings should be interpreted as describing the surveyed population rather than as weighted national estimates.

### Data collection

Data collection was conducted in five provinces of Burundi, namely Bujumbura, Gitega, Buhumuza, Burunga, and Butanyerera. These provinces reflect different geographical and socio-demographic contexts. Data collection took place in two phases. The first phase was conducted in Bujumbura from 29 March to 9 April 2025. The second phase covered the other provinces from 18 to 31 December 2025. This second phase was delayed due to funding and operational constraints.

Data were collected using a pretested structured questionnaire administered by trained interviewers. The questionnaire included five sections covering socio-demographic characteristics, mpox knowledge, perceptions, preventive practices, and health-seeking behaviours. Data were collected electronically using REDCap, with daily quality checks performed through data validation and consistency queries.

### Outcome definitions

Knowledge was assessed using 6 items covering knowledge of viral causes, animal/human susceptibility, personal risk awareness, and rejection of misconceptions regarding divine causes, government propaganda, and discrimination. Each correct answer was assigned one point and incorrect or ‘don’t know’ responses were assigned zero points. The total knowledge scores ranged from 0 to 6. Participants scoring 100% of the maximum score were classified as having adequate knowledge.

Preventive practices were assessed using 4 items related to hand hygiene, health professional consultation, isolation/testing when symptomatic, and seeking immediate healthcare advice. A composite practice score was generated using the same approach. Participants scoring ≥80% were considered to have adequate preventive practices.

### Data management and analysis

The dataset used for analysis comprised 5,177 records. Age eligibility was restricted to participants aged 15–100 years. Fourteen records had out-of-range age and were excluded. This left 5,163 participants with complete demographic data. For each regression model, records with missing values on any variable used in that model were further excluded. This included the outcome, all predictors, and the clustering variable. Missing data were handled using a complete-case approach. No imputation was performed. This resulted in a final analytic sample of 4,994 participants for all three outcome models.

Data were cleaned, managed, and analysed using Python (version 3.10.9). Data processing and recoding were performed using pandas and numpy, while statistical analyses were conducted using scipy and statsmodels. Descriptive analyses were conducted to summarise participant characteristics and key variables. Categorical variables were summarised using frequencies and proportions, while continuous variables were summarised using means and standard deviations (SD) or medians and interquartile ranges (IQR), as appropriate. Primary outcomes, i.e., mpox message exposure, comprehensive knowledge, and prevention and control practices, were summarised as proportions.

Prior to regression modelling, multicollinearity among candidate predictors was assessed using a pairwise correlation matrix computed with scipy. Cramér’s V was used for categorical variables, and Spearman’s rank correlation was used for continuous or ordinal variables. An association ≥0.60 was considered evidence of high collinearity, warranting removal of one variable from the pair; 0.40–0.59 was flagged as moderate. No pairs exceeded the high-collinearity threshold (maximum observed association: 0.36, between age and education level), so all candidate predictors were retained for modelling.

Regression models were fitted using statsmodels. Bivariate associations between each candidate predictor and each outcome (mpox message exposure, comprehensive knowledge, and prevention and control practices) were assessed using modified Poisson regression [18] with a log link and cluster-robust (sandwich) standard errors, clustered at the district level to account for within-district correlation. For the multivariable analysis, all candidate predictors were included simultaneously in a fully adjusted model for each outcome, regardless of their bivariate significance. Variables were selected a priori based on epidemiological relevance, biological plausibility, and previous knowledge of factors associated with mpox-related outcomes rather than statistical selection procedures. Adjusted prevalence ratios (aPRs) and 95% confidence intervals were reported.

## Results

### Demographic and comorbidities characteristics of study participants

Table 1 summarizes the demographic and health characteristics of study participants. A total of 5,313 individuals were enrolled, of whom 5,177 were included in the final analysis after excluding participants with missing data. Among participants with complete demographic information (n = 5,163), females represented 56.8% of the study population, and the median age was 34 years (IQR: 26–45 years). Most participants were married or living with a partner (72.5%), had primary education as their highest educational level (39.9%), and were mainly engaged in agriculture (64.4%). Self-reported comorbidities were reported by a minority of participants, including previous STI diagnosis (8.8%), HIV infection (5.7%), tuberculosis (2.3%), and malnutrition (1.2%).

**Table 1:** Frequency distribution of demographic and comorbidities characteristics of study participants.

Demographics	Frequency (%)N = 5,163
**Age in years**	
Mean ± SD	36.9 ± 13.8
**Sex**	
Female	2,930 (56.7%)
Male	2,233 (43.3%)
**Marital status**	
Married	3,750 (72.6%)
Single	1,063 (20.6%)
Widow(er)	256 (5.0%)
Separated	51 (1.0%)
Divorced	36 (0.7%)
Other	7 (0.1%)
**Education level**	
Primary	2,052 (39.7%)
Secondary	1,362 (26.4%)
No education	1,210 (23.4%)
Non-formal education	283 (5.5%)
Tertiary/University	255 (4.9%)
*Missing*	*1*
**Profession**	
Farmer	3,401 (65.9%)
Private	682 (13.2%)
Trading/Business	487 (9.4%)
Civil/public servant	302 (5.8%)
Unemployed	291 (5.6%)
**Religion**	
Christian	4,934 (95.6%)
Islam	200 (3.9%)
Others	24 (0.5%)
Traditional Worshipper	3 (0.1%)
*Missing*	*2*
**Income level**	
Less than 3,000 BIF/day	2,072 (40.1%)
3,000–30,000 BIF/day	1,516 (29.4%)
No income	1,383 (26.8%)
33,000–60,000 BIF/day	131 (2.5%)
>90,000 BIF/day.	34 (0.7%)
63,000–90,000 BIF/day	27 (0.5%)
**STI diagnosis**	
No	4,708 (91.2%)
Yes	455 (8.8%)
**HIV/AIDS diagnosis**	
No	4,867 (94.3%)
Yes	294 (5.7%)
*Missing*	*2*
**Tuberculosis diagnosis**	
No	5,032 (97.5%)
Yes	118 (2.3%)
*Missing*	*13*
**Malnutrition diagnosis**	
No	5,097 (98.7%)
Yes	60 (1.2%)
*Missing*	*6*
**Mpox Burden (District_Zone GRC)**	
High	3,961 (76.7%)
Low	1,057 (20.5%)
*Missing*	*145*

Note: N = 5,163 due to missing demographic information for 14 participants.

### Geographic distribution of the participants

The study included participants from all five provinces of Burundi: Gitega (n = 1,747; 33.7%), Bujumbura (n = 1,684; 32.5%), Buhumuza (n = 843; 16.3%), Burunga (n = 458; 8.8%), and Butanyerera (n = 445; 8.6%). The distribution of participants by district is shown in Fig 1. Ntahangwa health district had the highest number of participants (n = 419), followed by Kabezi health district (n = 339), while Nyabiranga/Mugongomanga health district had the lowest (n = 145).

**Fig 1:**
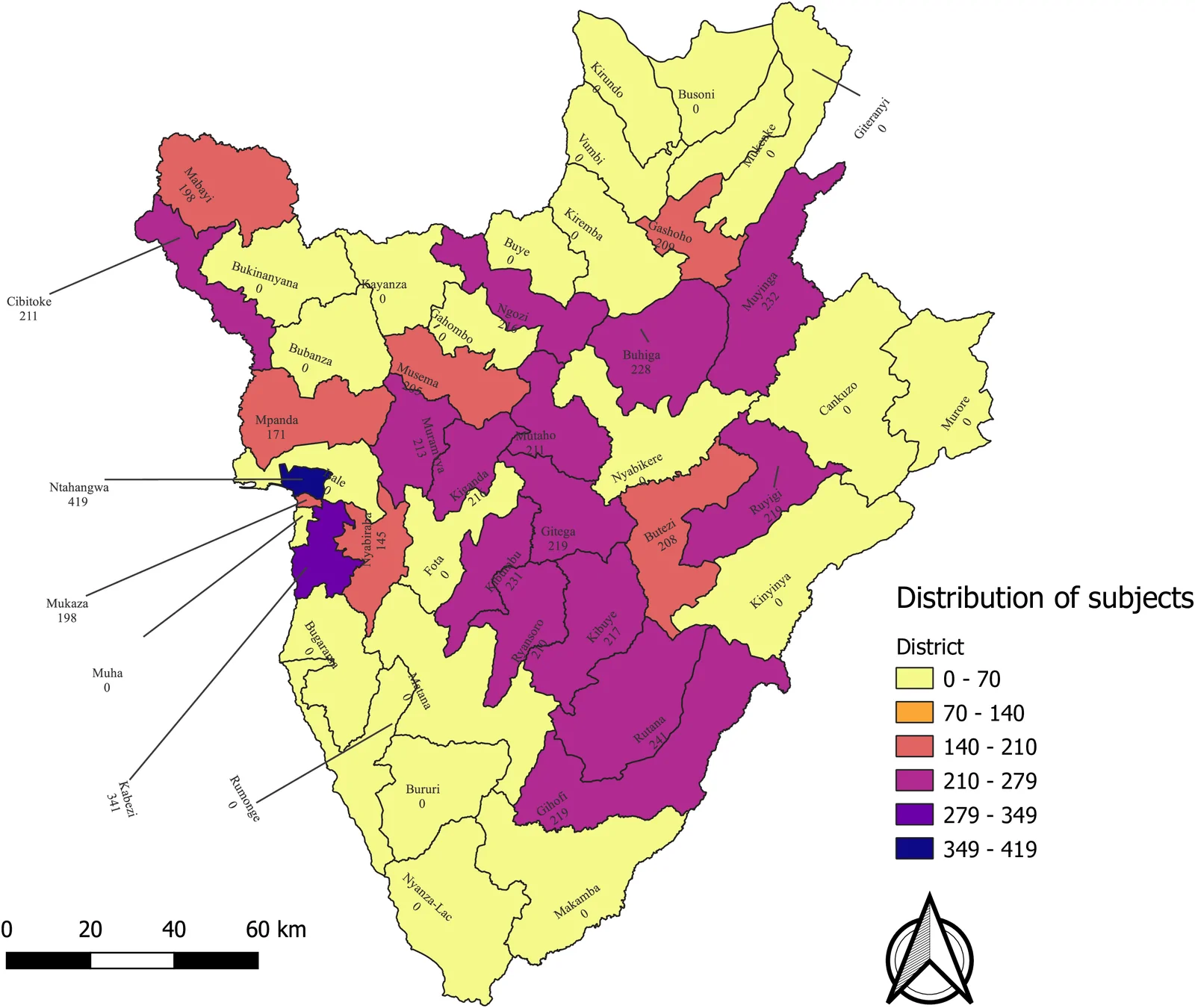
Geographic distribution of participants per health district in Burundi. The map presents the number of participants enrolled in each health district during the mpox survey. The distribution reflects the implementation of the survey and the geographic coverage achieved during the mpox outbreak response and should not be interpreted as population-weighted estimates.

### Knowledge of mpox causes, symptoms and transmission

Among the interviewed participants, awareness of mpox was high. Overall, 94.7% of participants reported having heard about mpox. Among participants aware of mpox, 52.0% believed the disease was caused by animals, whereas 21.4% attributed it to bacteria and 20.3% identified viruses as the causative agent. However, 24.5% did not know the cause of the disease. Recognition of symptoms was generally high. Skin rash was identified by 94.4% of p, followed by fever (84.3%) and headache (45.0%). Regarding transmission, 86.6% identified direct contact with infected persons, 62.3% identified kissing, and 42.7% recognized sexual contact as potential transmission routes.

### Perceived high-risk groups for mpox infection

Sex workers (98.6%), people with multiple or new sexual partners (98.3%), individuals who had intimate contact with an infected person (98.3%), and travellers to affected areas (97.7%) were the groups most frequently perceived as being at risk. Household members of an infected person were also commonly identified as vulnerable to infection (97.3%). Although recognition was somewhat lower for older adults (84.4%), long-distance drivers (86.8%), children (87.4%), hunters (87.7%), and people living with HIV/AIDS (88.4%), most participants still considered these groups to be at risk (Table 2).

**Table 2:** Participants perceptions of population groups at high risk of mpox infection.

	N	Yes % (n)	No % (n)
**Aged persons**	4894	84.4 (4129)	15.6 (765)
Long distance drivers	4893	86.8 (4249)	13.2 (644)
Children	4900	87.4 (4283)	12.6 (617)
Hunters	4902	87.7 (4301)	12.3 (601)
People living with HIV/AIDS	4899	88.4 (4331)	11.6 (568)
Health workers taking care of infected people	4900	91.9(4501)	8.1 (399)
People who prepare and transform bush meat	4899	93.0 (4556)	7.0 (343)
Homobisexual	4901	93.7 (4591)	6.3 (310)
Members of household of affected person	4898	97.3 (4767)	2.7 (131)
Travellers to affected zones	4899	97.7 (4787)	2.3 (112)
people with intimate contact with infected person	4902	98.3 (4818)	1.7 (84)
People with multiple sexual partners or new partners	4902	98.3 (4818)	1.7 (84)
Sex workers	4895	98.6 (4827)	1.4 (68)

### Information sources regarding mpox

Radio was the most frequently reported source of mpox information (82.0%), followed by social media (21.1%), and television (11.7%) (Fig 2A). While radio was reported by the majority 87.0% (n = 4504) to be the most trusted source of information, religious leaders and health professionals were reported by 20.4% and 8.0 respectively to be the trusted source of information (Fig 2B). However, 95.0% of participants expressed a desire for additional information regarding mpox.

**Fig 2:**
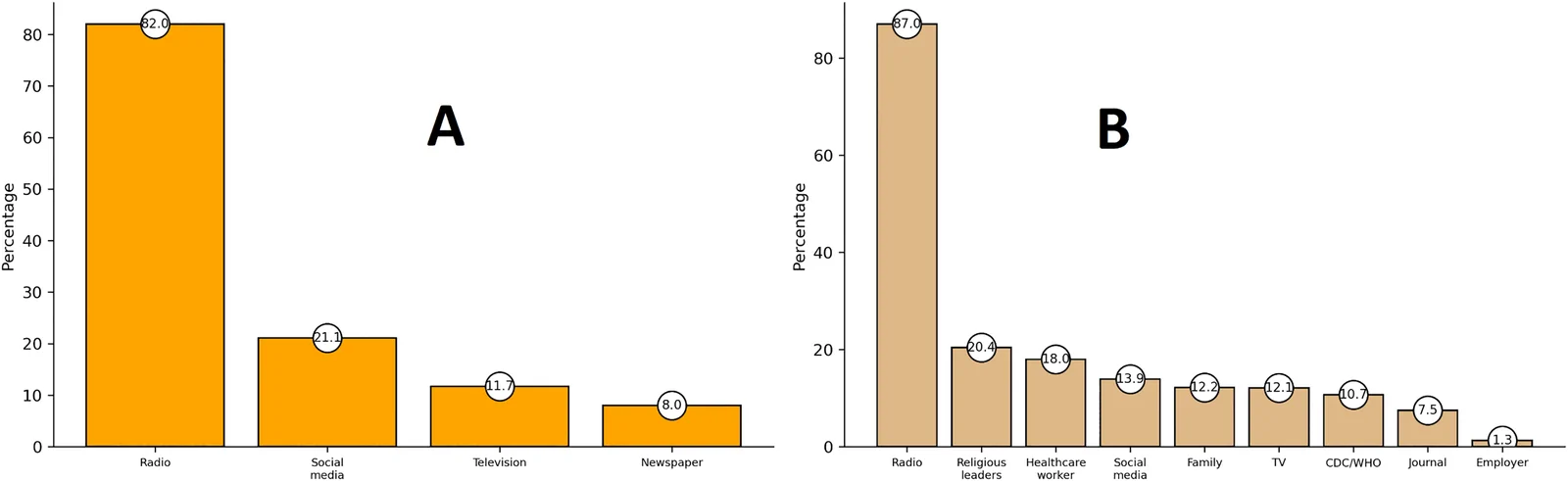
Distribution of the participants based on the information source (A) and trusted information sources (B).

### Perceptions toward mpox prevention and control

Table 3 presents participants’ perceptions regarding key mpox prevention and control messages. Overall, participants showed good recognition of important information related to mpox. Most participants considered mpox a serious disease (96.8%) and recognized the importance of preventive measures, including hand washing (91.9%), disinfection of surfaces (91.0%), and vaccination (87.9%). Most participants also recognized that mpox can be transmitted from animals to humans and through sexual contact.

**Table 3:** Perceptions toward mpox prevention and control.

	N	Agreed % (n)	Uncertain % (n)	Disagree % (n)
Mpox is a serious disease	5169	96.8 (5005)	2.0 (103)	1.1 (60)
Mpox can be prevented	5169	92.4 (4777)	6.9 (355)	0.7 (37)
Mpox in animals can cause disease in humans	5169	91.8 (4745)	7.0 (362)	1.2 (62)
Processing or eating bush meat can predispose to mpox infection	5169	89.1 (4608)	8.9 (462)	1.9 (99)
Sex with an mpox case can cause mpox	5169	92.9 (4801)	5.9 (304)	1.2 (62)
Hand washing can reduce transmission of Mpox	5169	91.9 (4751)	6.5 (337)	1.6 (81)
Mpox is a curse from God and is healed by prayers	5163	18.4 (950)	15.8 (815)	65.8 (3398)
Vaccination against mpox can prevent the disease	5158	87.9 (4534)	9.8 (505)	2.3 (119)
Washing/disinfecting surfaces reduces transmission	5152	91.0 (4690)	7.4 (383)	1.5 (79)
Mpox disease is Government propaganda, it’s not real	5152	10.8 (558)	10.9 (560)	78.3 (4034)
It is right to discriminate against mpox cases	5152	40.7 (2097)	10.7 (550)	48.6 (2505)

However, some misconceptions and negative attitudes were still observed. Some participants continued to associate mpox with beliefs such as a curse that could be treated through prayer, and a proportion considered mpox as government propaganda rather than a real disease. Attitudes towards discrimination were also concerning, as a substantial proportion of participants supported discriminatory views towards people affected by mpox. These findings highlight the need to maintain community engagement and risk communication activities during mpox response efforts.

### Vaccine awareness and acceptance

Among those asked about vaccine availability, 86.1% reported that they were not aware of the existence of a vaccine against mpox. A high proportion of participants reported willingness to be vaccinated. Overall, 76.5% stated that they would definitely accept vaccination if it were available, while 17.7% reported that they would probably accept it. Nearly 90% of participants believed that their family members, friends, religious leaders, and community leaders would support vaccination. Similarly, about 90% reported being very sure or almost sure that the vaccine would be safe.

### Factors associated with exposure to mpox-related messages in Burundi

Factors associated with exposure to mpox-related messages are presented in Fig 3. Overall, radio was the most frequently reported source of mpox information (82.0%), reflecting its important role in community communication during the outbreak. After adjustment, exposure to mpox-related messages was associated with several socio-demographic characteristics. Participants with higher education levels were more likely to report exposure compared with those without formal education, including those with primary education (aPR = 1.05, 95% CI: 1.02–1.07), secondary education (aPR = 1.05, 95% CI: 1.02–1.09), and tertiary/university education (aPR = 1.07, 95% CI: 1.03–1.11). Exposure was also higher among some occupational groups, including civil/public servants and private-sector workers, compared with farmers. Differences were observed according to marital status, income level, and religion, with participants reporting traditional or other religions showing higher exposure to mpox-related messages. No significant association was found with sex, district mpox burden, or reported comorbidities, including STI, HIV infection, tuberculosis, and malnutrition.

**Fig 3:**
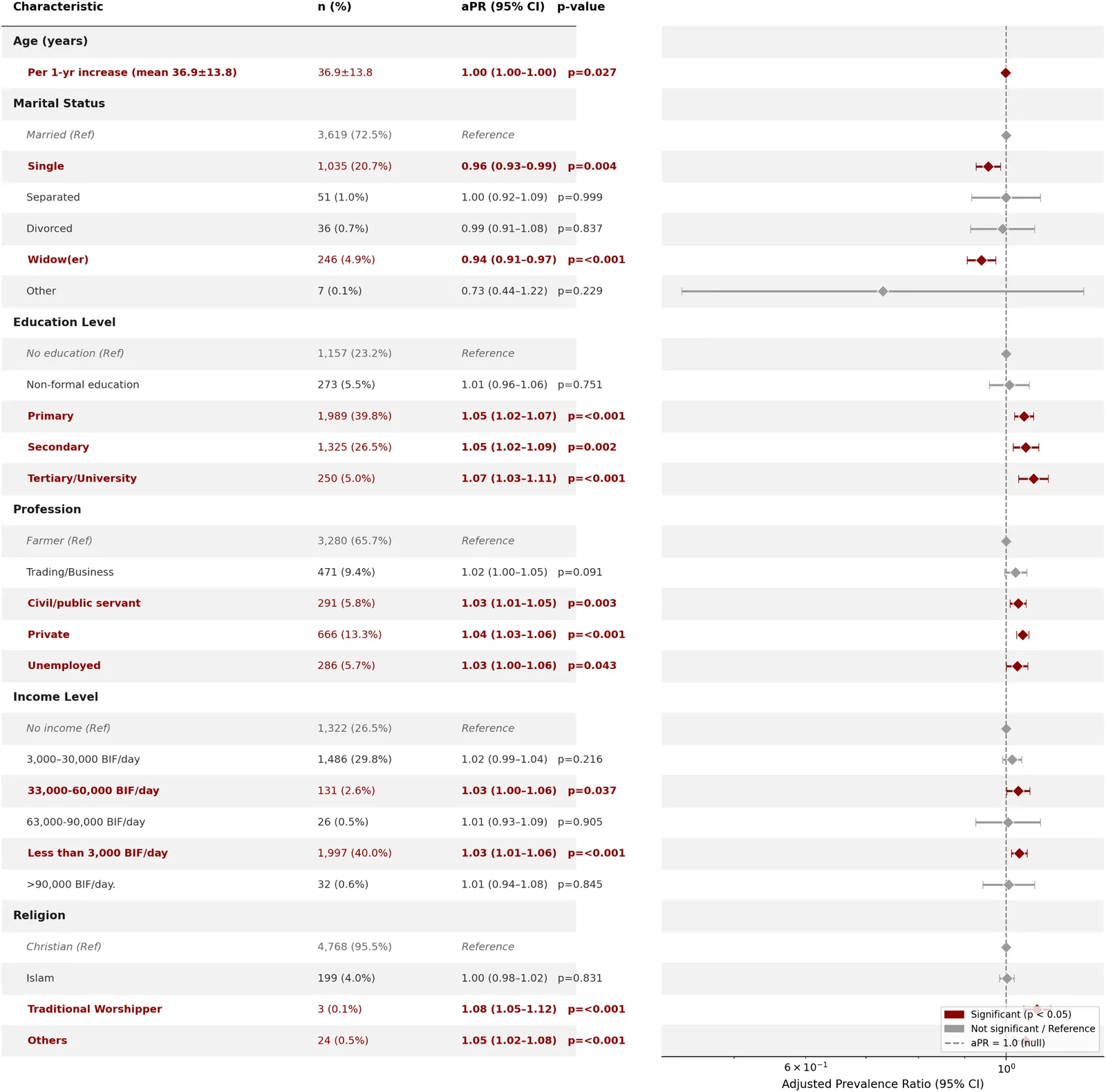
Factors associated with exposure to mpox messages in Burundi.

### Factors associated with overall mpox knowledge in Burundi

Factors associated with overall mpox knowledge are presented in Fig 4. Overall mpox knowledge varied according to socio-economic and demographic characteristics. Age was negatively associated with adequate mpox knowledge, although the effect was small (aPR = 0.99, 95% CI: 0.99–1.00). Compared with participants without income, those reporting higher income levels were more likely to have adequate mpox knowledge, with the strongest association observed among participants earning more than 90,000 BIF/day (aPR = 5.13, 95% CI: 3.82–6.88). Differences were also observed according to occupation, with unemployed participants showing higher knowledge compared with farmers, while traders/businesspersons and private-sector workers were less likely to have adequate knowledge. Muslim participants were more likely to have adequate mpox knowledge compared with Christian participants (aPR = 1.49, 95% CI: 1.09–2.03). No significant associations were observed with education level, marital status, district mpox burden, or reported STI, HIV infection, tuberculosis, or malnutrition after adjustment.

**Fig 4:**
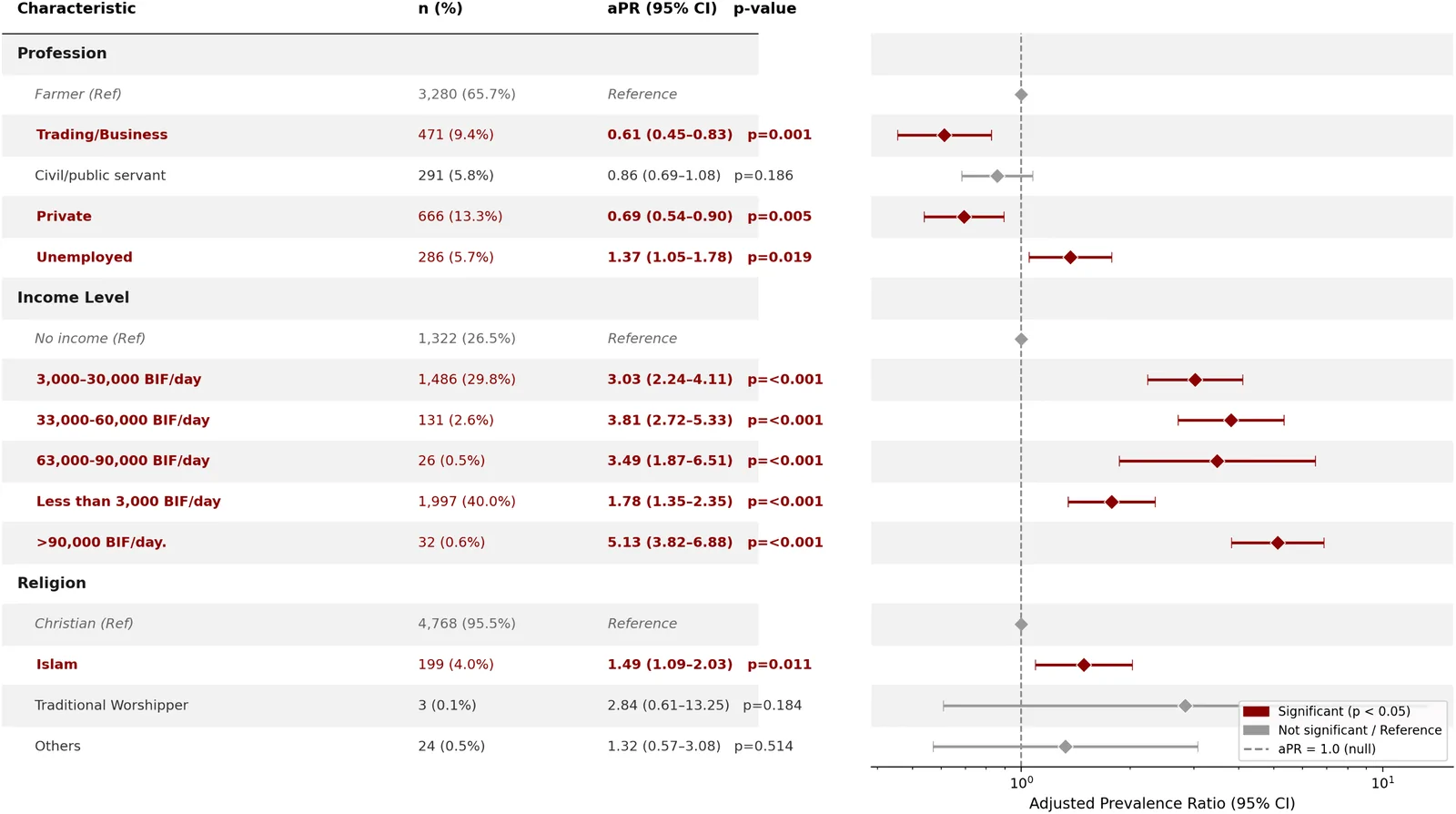
Factors associated with overall mpox knowledge in Burundi.

### Factors associated with mpox prevention and control practices in Burundi

Factors associated with mpox prevention and control practices are presented in Fig 5. Adequate prevention and control practices were associated with several socio-demographic and knowledge-related factors. Participants with secondary education were more likely to report adequate practices compared with those without formal education (aPR = 1.13, 95% CI: 1.01–1.25). In contrast, widowed participants were less likely to report adequate practices compared with married participants (aPR = 0.77, 95% CI: 0.67–0.89). Participants living with HIV infection were more likely to report adequate practices (aPR = 1.32, 95% CI: 1.01–1.72), while those exposed to mpox-related messages showed lower reported prevention and control practices after adjustment (aPR = 0.79, 95% CI: 0.70–0.90). Importantly, participants with adequate comprehensive knowledge of mpox were more likely to report adequate prevention and control practices (aPR = 1.33, 95% CI: 1.23–1.43). No significant associations were observed with age, sex, occupation, district mpox burden, or reported STI, tuberculosis, and malnutrition after adjustment.

**Fig 5:**
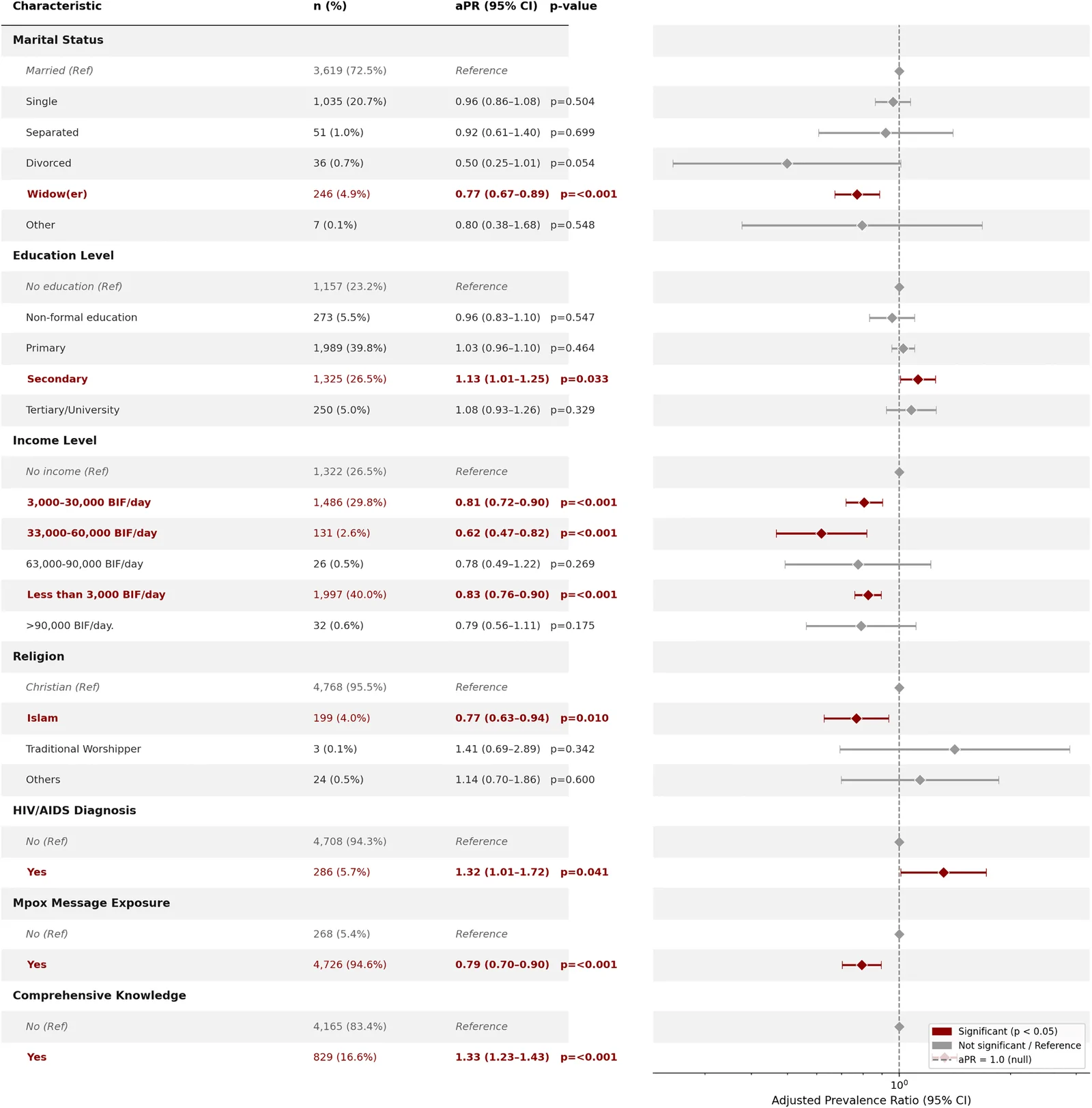
Factors associated with mpox prevention and control practices in Burundi.

## Discussion

This study provides evidence from a nationwide survey on mpox awareness, knowledge, perceptions, and prevention and control practices in Burundi. Overall, awareness was high, but important gaps remain in knowledge and practice.

Awareness of mpox was high in the study population, which may reflect the extensive risk communication activities implemented during the outbreak response in Burundi. Following the detection of the first mpox cases in July 2024, the Ministry of Public Health, in collaboration with partners including WHO, UNICEF, and Africa CDC, implemented community sensitization activities, media campaigns, and targeted health education interventions across the country [19–21]. However, despite this high level of awareness, exposure to mpox-related information was not equally distributed among participants, with differences observed according to several sociodemographic characteristics. These findings suggest that while national communication activities reached a large proportion of the population, some population groups may require more targeted approaches to ensure equitable access to reliable mpox information.

Despite high awareness, important knowledge gaps and misconceptions remained, particularly regarding the causes and transmission routes of mpox. Although most participants recognized animal-to-human transmission, some participants attributed mpox to bacteria or were uncertain about its cause. Similar misconceptions have been reported in other African countries during recent mpox outbreaks, indicating that increased awareness does not always translate into complete understanding of disease transmission [22].

Recognition of common symptoms was generally high, particularly for skin rash and fever, which is consistent with findings reported from Nigeria and the Democratic Republic of Congo during previous outbreaks [23,24]. However, knowledge of some transmission routes, including sexual transmission and kissing, was lower. This pattern has also been described elsewhere and may reflect the evolution of mpox epidemiology, where different transmission patterns emerged during recent outbreaks [25]. This is consistent with epidemiological evidence showing that close physical contact remains an important driver of transmission [8,9] and may also reflect the awareness activities implemented during the outbreak response [4].

Participants with adequate mpox knowledge were more likely to report adequate prevention and control practices. This association suggests that access to accurate information may influence the ability of individuals to adopt preventive behaviors. Similar findings were reported among university students in Ghana, where higher mpox knowledge and more positive attitudes were associated with better preventive practices using structural equation modelling. These findings support the importance of improving community knowledge as part of mpox preparedness strategies [26]. Differences observed between social groups may reflect unequal access to health information, education, and health services. In Burundi, people with higher educational levels or formal occupations may have greater opportunities to receive and understand health messages through workplaces, media, and other information channels. Conversely, populations with fewer educational and economic resources may require more adapted communication approaches using local languages, community health workers, and trusted community structures. Similar associations between socioeconomic characteristics and mpox knowledge or perceptions have been reported in other African settings [26].

The study highlights a dominant reliance on radio as the main information source (82%) and the most trusted channel (87%). Radio was the most widely used and trusted source because it is accessible across most parts of Burundi, particularly in rural areas. Health workers and community health workers also played an important role consistent with evidence that interpersonal communication is critical in outbreak response [27]. In the Burundian context, the community health workers are at the base of the public health system, forming its primary operational layer. In contrast, social media was less trusted and less used, consistent with concerns about misinformation in online platforms [28]. Exposure to mpox-related messages was not equally distributed among participants. Individuals with higher education and those working in formal sectors had greater exposure, suggesting differences in access to health information. Single and widowed participants had lower exposure, highlighting the need for communication strategies that also reach socially vulnerable groups.

Interestingly, exposure to mpox-related messages was associated with lower preventive practices. The negative association between exposure to mpox-related messages and preventive practices should be interpreted with caution. This finding should not be interpreted as evidence that communication campaigns reduce preventive behaviours. One possible explanation is that communication activities may have been more focused on communities with higher risk or lower preventive practices. In addition, receiving information does not always mean that people understand or apply the recommended measures. Future longitudinal studies are needed to better understand the relationship between communication exposure and preventive behaviors.

Preventive behaviors were generally good, especially regarding hygiene practices, with more than 92% of respondents recognizing handwashing and 91% recognizing surface disinfection as protective measures. This may partly reflect the extensive awareness and community sensitization activities implemented during the outbreak response. However, important socio-economic differences were observed. Participants in some income categories were less likely to report adequate prevention and control practices compared with those reporting no income, suggesting that socioeconomic conditions may influence the ability to apply preventive measures. Similarly, higher income was associated with better knowledge of mpox, highlighting inequalities in access to health information and educational resources. These findings are consistent with previous evidence showing that social and economic conditions strongly influence health behaviors and access to health information [13,27].

Widowed participants were less likely to report adequate preventive practices, which may be related to lower social support. In contrast, people living with HIV were more likely to adopt preventive measures. This association may be related to more frequent interaction with health services and exposure to health education through HIV care programs, although further studies are needed to confirm this explanation [29]. The differences observed across income and social groups suggest that the adoption of preventive practices may also depend on people’s living conditions and available resources.

Regarding vaccination, the high willingness to accept vaccination (76.5% definitely willing) is consistent with global studies reporting moderate-to-high mpox vaccine acceptance when risk perception is high [30–33]. Nearly 90% of participants trusted vaccine safety, which is encouraging for future immunization strategies. However, 86.1% were unaware of the existence of a vaccine, indicating a major communication gap. This discrepancy between willingness and awareness suggests that lack of information about vaccine availability may be an important barrier, rather than vaccine hesitancy alone [30].

The findings can be explained using the socio-ecological framework, which shows that mpox prevention is influenced by different levels. At the individual level, people with better knowledge were more likely to use preventive measures. At the community and interpersonal levels, health workers, community health workers, family members, religious leaders, and radio were important sources of information. At the structural level, people with low income had less knowledge and poorer preventive practices. Overall, improving mpox preparedness needs combined actions: better education, strong communication through trusted sources, and support for poorer groups. These findings have two important public health implications. First, health messages should be shared through trusted channels like radio and community health workers. Second, special attention is needed for economically vulnerable populations, who may have less access to health information and preventive resources.

### Limitations

This study has some limitations. Data were collected in two phases, and changes in the outbreak situation or public health communication during this period may have influenced participants’ responses. The information was self-reported and may be affected by recall and social desirability bias. In addition, willingness to receive the mpox vaccine was assessed under a hypothetical situation because the vaccine was not available in Burundi at the time of the study. Sampling weights were not applied, and therefore the findings should be interpreted within the context of the surveyed population. Also, knowledge and preventive practice were measured using composite scores, which may have resulted in some misclassification of participants. Despite these limitations, the study provides important information on mpox preparedness among the surveyed population in Burundi.

## Conclusion

This study provides the first nationwide assessment of behavioral and social determinants of mpox preparedness among surveyed populations in Burundi. Even if most people know about mpox and are willing to accept vaccination, important gaps in knowledge and differences between income groups still exist. Many people also do not know that a vaccine exists, even if they are willing to take it. To improve preparedness, it is important to strengthen health messages through radio and community workers and to reduce inequalities between rich and poor groups.

## Supporting information

S1 File Study protocol and qualitative checklist tool for assessing socio-ecological and behavioral drivers of mpox disease in Burundi. This file contains the study protocol, including the study background, objectives, methodology, sampling strategy, data collection procedures, ethical considerations, informed consent process, and the qualitative checklist tool used to explore community perceptions, behaviors, stigma, prevention practices, and vaccine perceptions related to mpox disease.
(DOCX)

S2 File Field implementation procedures for community-based household surveys. This file describes how the field teams conducted the survey, including preparation of the teams, identification and selection of households, contact with households, participant recruitment and consent, questionnaire administration, supervision, data quality checks, confidentiality, and reporting of field problems.
(DOCX)
