# Supplementary material for: Community knowledge and perceptions of social, ecological, and behavioural risk factors for mpox in Burundi: A nationwide cross-sectional survey
Source: PLOS Glob Public Health. 2026 Oct 27;6(10):e0007383. doi: 10.1371/journal.pgph.0007383 (PMC13712512; doi:10.1371/journal.pgph.0007383)
Supplement: Supplementary file 1 [file pgph.0007383.s001.docx]

**UNDERSTANDING THE SOCIO-ECOLOGICAL AND BEHAVIORAL DRIVERS OF MPOX DISEASE IN AFRICA**

**SCIENCE AND INNOVATION DIRECTORATE OF THE AFRICA CDC**

**IN COLLABORATION WITH**

**AFRICA UNION MEMBER STATES NATIONAL PUBLIC HEALTH INSTITUTES/ MINISTRIES OF HEALTH**

**AND**

**INFECTIOUS DISEASE INSTITUTE, MAKERERE UNIVERSITY, UGANDA/ AFRICAN FORUM FOR RESEARCH AND EDUCATION IN HEALTH, GHANA**

**SEPTEMBER 2024**

Contents

1.0 Background	3

1.1	5Specific Objectives	5

Research Questions	6

2.0 Methods	6

2.1 Study Design and Duration	6

2.2. Study Region	6

2.3 Study Population	7

2.4 Sample Size	7

2.5 Sampling Techniques	7

2.6 Recruitment of Participants	7

2.7 Inclusion and Exclusion Criteria	8

2.8 Data Collection	8

2.9 Data Management and Analysis	8

3.0 Ethical Considerations	9

4.0 Administrative Considerations	9

4.1 Data Management Systems	9

5.0 Schedule	10

6.0 Budget	11

8.0 References	11

Qualitative Checklist Tool	12

# 1.0 Background

Mpox (formerly known as monkeypox) is caused by mpox virus (MPXV), a zoonotic disease which causes small-pox like infections first reported in humans in the Democratic Republic of Congo (DRC) in 1970 (1). MPXV emerged as an important orthopox virus in public health following the successful eradication of smallpox and cessation of the global smallpox vaccination program (2, 3). The 2022-2023 global Mpox epidemic resulted in a cumulative total of 93,921 laboratory-confirmed cases, including 179 deaths, from 117 countries, of which 15 were African countries (4).

On 13 August 2024, the Director General of Africa CDC, following the guidance of the Emergency Consultative Group (ECG), declared the Mpox outbreak a Public Health Emergency of Continental Security (PHECS) (5). Subsequently, on 14 August 2024, the World Health Organization (WHO) Director-General also declared the Mpox outbreak a Public Health Emergency of International Concern (PHEIC) due to the increase in Mpox cases in the DRC and its expansion to neighboring countries, per the International Health Regulations (2005) (6).

This spread presents a public health risk to other Member States and requires a coordinated international response. As of 18 August 2024, 12 countries had reported 3,562 confirmed cases, and 26 deaths. The three countries with the majority of the cases in 2024 are the DRC (n = 3,235), Burundi (n = 153), and Central African Republic (n = 45). The spread of cases in the Democratic Republic of Congo is attributed to two distinct outbreaks - the spread of MPXV clade Ia in Equateur and endemic regions of the country and the emergence of a new MPXV clade (clade Ib) in the South and North Kivu provinces.

The sudden appearance of a multi-country outbreak of Mpox disease in 2022, which reached over 110 countries, and the human-to-human spread of MPXV through sexual and social networks around the world have highlighted the need to better understand human-to-human transmission and factors that contribute to the emergence and re-emergence of this virus over time.

To date, it is understood that human-to-human transmission can occur through direct contact with infectious skin or mucocutaneous lesions; this includes face-to-face, skin-to-skin, mouth-to-mouth, or mouth-to-skin contact and respiratory droplets (and possibly short-range aerosols requiring prolonged close contact) (7). The current global outbreak has been associated with sexual activity in some networks; the epidemiological analysis of the 2022 global Mpox epidemic demonstrated a high representation of infections in males and primarily those who identified as gay, bisexual, or other men who have sex with men, as well as higher risks of infection associated with HIV infection status (8-10). The presence of lesions on scrotal tissues, groin, or buttocks had previously been noted in reports from 1988 and 2005 (11), raising the possibility that human infection from intimate sexual activity may have already been a mode of transmission in the past.

Descriptions of the disease in Nigeria from 2017-2018 also suggest that intimate sexual contact could have been associated with infection and transmission. However, it was not recorded as a mode of transmission at the time (12, 13). While within Clade I infections, historically reported from DRC, congenital infection during pregnancy was documented, and the mode of transmission was presumed to be by direct contact (11). More recently, researchers depicted a case series review of four pregnant women who have Mpox infection, among whom only one had a live birth following vertical transmission of MPXV, and of the rest, at least one of the stillborn fetuses had evidence of Mpox lesions (14).

This emergence and re-emergence of new and old infectious diseases has widely been linked to the socio-ecological model (15). The socio-ecological model provides a framework for exploring the interaction of individuals, their health, and physical and social environments at multiple levels of a health problem and interdependence. At the individual level, poverty, vulnerability, malnutrition, and poor healthcare behavior are more prevalent in low-income regions, including sub-Saharan Africa(9, 16-18). At the interpersonal level, community factors, such as social networks made of family, friends, or colleagues, provide support. Institutional factors such as formal or informal organizations may have rules or expectations that impact health behaviors. Laws, policies, or regulations may promote or inhibit certain health practices that impact disease management, control, or prevention.

Thus, it is critical to identify the socio-ecological and behavioral determinants that underscore the Mpox pandemic. Understanding these social drivers of the emergence and re-emergence of Mpox disease is pertinent for containing the outbreak at source, preventing spread, and understanding evolution patterns of similar viruses with the potential to cause future outbreaks. Understanding the prior emergence of MPXV in African countries and newly affected countries in 2024 is critical to designing appropriate interventions that will reduce the public health impact of Mpox disease in the most affected countries.

There is an urgent need for evidence and contextual lessons to inform risk communication and community engagement to build outbreak preparedness, management, and mitigation activities within at-risk communities. Such interventions will preferentially target at-risk and marginalized populations in endemic countries but equally on all citizens on the continent independent of economic status.

Thus, we will assess Mpox risk perception and identify individual, social, structural, and environmental determinants based on the socio-ecological model of infection and vaccination uptake perception to strengthen community engagement and preparedness, focusing on all African Union Member States (AU MS). The study will explore individual-level, environmental, societal, and national structural drivers.

## 1.1 Objectives

### General Objective

To assess the state of knowledge, attitudes, and practices, including socio-ecological and behavioral determinants of health considerations, regarding MPXV acquisition in AU MS

### Specific Objectives

1. To identify the determinants of the Mpox disease spread and modes of transmission.
2. To understand the community’s knowledge and sources of information on Mpox disease.
3. To assess factors that contribute to stigma and discrimination to Mpox disease.
4. To describe attitudes towards MPXV acquisition and its preventive measures.
5. To describe the practices, including treatment-seeking behavior for Mpox disease.
6. To understand the barriers and facilitators to Mpox vaccine uptake
7. To determine the association between Mpox disease and comorbidities

### 1.2 Research Questions

1. What is the consensus of evidence about Mpox disease determinants and modes of transmission?
2. What do the communities know about Mpox disease? What sources of information on Mpox exist in the community?
3. What are the factors contributing to the stigma and discrimination of affected individuals?
4. What are communities’ perceptions about Mpox disease and its preventive measures?
5. What practices, including health-seeking behaviors, are communities carrying out to protect themselves from Mpox disease?
6. What are the barriers and facilitators to Mpox vaccine uptake, and recommend strategies for prioritizing and encouraging vaccination within the community?
7. What comorbidities are associated with Mpox disease?

# 2.0 Methods

## 2.1 Study Design and Duration

This will be a multicentric, multicountry descriptive cross-sectional study using a mixed-method approach. It will apply a socio-ecological system (SES) framework analysis as a lens to understand the multi-level factors associated with the Mpox disease outbreak in selected AU MS. The study will be conducted over two months from September to October 2024.

## 2.2. Study Region

This study will be conducted in all 55 AU MS in a phased model. However, for this first phase, we will include category 1 member states (DRC, Burundi, Central Africa Republic (CAR), the Republic of South Africa, and the Federal Republic of Nigeria), in addition to Kenya and the Republic of Congo in Category 2 (appendix 5.1).

## 2.3 Study Population

**Qualitative study:**

The qualitative study population will include key informants such as MoH experts, health workers, traditional leaders and healers, religious leaders, community leaders. The in-depth interview participants will include Mpox-affected individuals, their contacts and community members.

**Quantitative Study:**

Interviews will be conducted for Mpox disease patients, caregivers, household members or contacts, college-going students in urban areas, and those out of school in rural areas.

## 2.4 Sample Size

**For the qualitative study**: a minimum of 45 participants will be selected for the key informant and in-depth interviews in each Member State ensuring maximum variation by gender, region and category

**For the quantitative study**:

The sample size will be determined using the sample size formula for cross-sectional study: (Kirkwood and Sterne, 2003)

n =	(Z_1-α/2_+ Z_1-β_)^2^ [P(1-P_1_)]

(d)^2^

where:

n	=	Minimum sample size for each group

Z_1-α/2_ = Standard normal deviate corresponding to the probability of type I error

(α) at 5% =1.96.

Z_1- β_	=	Standard normal deviate corresponding to the probability of making	type II error (β) of 20%, Power at 90% = 1.28

P	=	Prevalence of knowledge of Mpox in Nigeria is 29% (19).

n	=	(1.96 + 1.28)^2^ (0.29 [1-0.29])

(0.05)^2^

n =	865

With a non-response rate of 10%, the sample size will be 951.

**Design effect**

Country reporting cases at least 1 sample size 951 *2= 1902

Countries with cases	<100 sample size 951 *3 = 2853

Countries with cases >100 - <200 sample size 951 * 4= 3804

Countries with cases >200 - <300 sample size 951 * 5= 4755

Countries with cases >300 - <400 sample size 951 * 6= 5706

Countries with cases >400 - <500 sample size 951 * 7= 6657

Countries with cases = or >500	951 * 8= 7608

| Countries | Mpox cases in August 2024 | Minimum sample size |
| --- | --- | --- |
| DRC | 3244 | 7608 |
| CAR | 45 | 1902 |
| Burundi | 231 | 4755 |
| Nigeria | 41 | 1902 |
| South Africa | 24 | 1902 |
| Kenya | 2 | 1902 |
| Congo | 23 | 1902 |

## 2.5 Sampling Techniques

**Qualitative:** Qualitative participants will be selected using purposive sampling for each participant category to observe representation and maximum variation.

**Quantitative**: A multistage sampling technique will be employed to select participants to ensure representation across different levels of the Mpox burden in the country.

Stage 1: Selection of States/Provinces

Target Population: States/provinces with reported Mpox cases in 2024.

Sampling Method: Simple random sampling (SRS) will be used to select 50% of all affected states/provinces in each country.

At least 75% of these states/provinces will come from those with the highest Mpox burden, while 25% of the states/provinces will come from those with a low Mpox burden, using simple random sampling.

Stage 2: Selection of Local Government Areas/District

Target Population: LGAs/Districts within the selected states/provinces.

Sampling Method: SRS will be used to select 50% of LGAs/Districts in each selected state.

Among the selected LGAs, 75% will come from those with a high burden, and 25% from those with a low burden, using SRS.

Stage 3: Selection of Communities/settlements

Target Population: Communities within the selected LGAs/Districts.

Sampling Method: One affected community/settlement will be selected using SRS from the affected LGAs/Districts.

If the required sample size is not reached in the first community, the next contiguous community will be considered till we have the required sample size.

Note that probability proportionate to the size of each community or settlement will be used to determine the sample size for each based on the total sample size for the country.

Stage 4: Selection of Households and Adults

Target Population: Households within the selected communities.

Sampling Method: SRS will be used to select households with the starting point determined by spinning a bottle at the community center.

One eligible adult in each household will be included, based on predefined inclusion criteria. In households with multiple eligible adults, balloting (SRS) will be used to select the respondent.

The use of clusters by treating each community or settlement as a cluster is a practical way to manage the study and ensure efficient data collection. This cluster-based approach helps to focus on community-level insights while maintaining randomness and representation. Each community/settlement selected will be regarded as a cluster.

In each district, the district coordinator and team supervisors will first engage the district health teams and local community leaders before data collection to make them aware of the activity and reduce the risk of community suspicions and/or misinformation regarding its purpose.

## 2.6 Recruitment of Participants

Participants will be recruited at the first clinical visit following presentation to a healthcare center for suspected Mpox disease. Recruitment will be facilitated through partnerships with local community-based organizations. The household contacts presumably will not have signs of illness at recruitment.

## 2.7 Inclusion and Exclusion Criteria

Inclusion criteria will be defined by participants' willingness and consent to participate in the study, and participants' present at the healthcare facilities, isolation centers, camps, schools, colleges, and families of those infected within the community at the time of the study. Respondents between 15-17 years are also eligible. Exclusion criteria will include participants who are unwilling to participate and declined consent.

## 2.8 Data Collection

Data will be collected using a mixed approach for qualitative and quantitative data.

**Qualitative data collection**

Phenomenology theory will be used to collect data on lived experiences and personal perceptions from people affected by the Mpox who are the infected, contacts or household members and community members. Key informants will further share experiences based on collective observations and interpretations made of the subject of study in the different contexts.

Interview guides will be pretested to ensure that they are relevant to the local context of each member state. The adopted interview guides will be used to collect information from the participants.

Study participants will be requested for an appointment and a convenient schedule and venue will be agreed upon prior to the interview. Interviews will be recorded on an audio device and notes will be written. The recordings will be downloaded in less than 24 hours to a secure laptop with restricted access to ensure confidentiality and minimize data loss. For instances where participants decline their voices to be recorded, only written notes will be used for analysis.

Qualitative data collection

Quantitative data will be collected using interviewer-administered pretested questionnaires. The quantitative questionnaire will consist of five parts: 1) Socio-demographics; 2) Mpox knowledge and mode of transmission; 3) Potential MPXV acquisition activities; 4) Mpox risk perception across age and gender; 5) Practices around Mpox perception and treatment including health-seeking behavior, based on the socio-ecological model; 6) Comorbidity factors (nutrition status, HIV, Tuberculosis, NCDs etc).

**Data entry**: Trained data collectors will use digital recorders or personal digital assistants (PDAs) to capture data from the qualitative interviews, which will then be transferred for safe storage in a folder on a computer.

Quantitative data will be collected electronically and entered in real time into REDCap software using tablets. Each interview will last approximately 30 to 45 minutes. Phenomenology theory will collect data on lived experiences and personal perceptions of the diseases from people affected by Mpox who are the infected, contacts or household members, and community members. Key informants in this area will further share experiences based on collective observations and interpretations of the subject of study in different contexts.

**Pretesting**: All data collection tools will be pretested to ensure validity, suitability, and reliability before actual data collection.

## 2.9 Data Management and Analysis

**Qualitative data:** The audios will be transcribed verbatim, and quality assurance will be done to ensure all information was captured. The data will be analyzed using both a deductive and inductive approach informed by the socio-ecological model. A minimum of two coders will analyze a quarter of the overall transcripts through content analysis, ensuring that the selected transcripts represent each respondent category. A coding framework will be generated after the coders reach a consensus on how the codes align with the attributes of the socio-ecological model. This framework will present a broad understanding of the socio-behavioral drivers of Mpox disease across individual, interpersonal, community, and societal levels. The framework will be entered in NVivo analysis software for a systematic analysis of all the transcripts.

**Quantitative data:** The quantitative data will be cleaned by data querying in REDcap to ensure completeness and accuracy. Clean data will then be exported to STATA for analysis. As appropriate, data will be described using frequencies and percentages for continuous and categorical variables. The Chi-square test will be used in bivariate analyses to examine socio-demographic factors, knowledge, attitude and practice levels. Regression models can also be used.

## 3.0 Ethical Considerations

**Good clinical practice and human subjects protection**

This study will be conducted in compliance with the Declaration of Helsinki and Good Clinical Practice Guidelines in full conformity with the principles of the Belmont Report: Ethical Principles and Guidelines for the Protection of Human Subjects of Research of the National Commission for the Protection of Human Subjects of Biomedical and Behavioral Research (April 18, 1979) and codified in 45 CFR 46, 21 CFR 312, and ICH E6; 62 Federal Regulations 25691 (1997). Each participating institution will hold a current FWA issued by the Office for Human Research Protection OHRP. All key study staff will be trained and certified in Good Clinical Practice. Both human and non-human research determination will be provided to guide ethical clearance in the respective member states. Ethical clearance will be provided by the Africa CDC continental Ethical Review Board (ERB), including local authorizations from Ministries of Health and Administrators at district hospitals and health facilities. National ethics committees will provide permission for the studies. Relevant national institutions will provide their approval for the study.

**Participants informed consent**

All participants approached for the survey will need to provide individual written/signed consent for the interview to be initiated. Respondents between 15-17 years are essential to include in the survey because they make up a substantial proportion of the at-risk age group for Mpox and carry significant risk for contracting Mpox. For this group of target participants ages 15-17, consent shall be sought from one of the parents. If none of the parents is available, consent from the child’s guardian or household head will be sought. In addition, permission will be sought from the child directly. It should be noted that a child’s dissent will take precedence over consent from his/her parent or guardian.

If a participant cannot read and write, eligible participants will be asked to sign the consent form with their thumbprint. No interview will be conducted without the prospective participant's written or thumbprint-signed consent. Each participant must sign two copies of the consent form – one will be left with the participant, and the survey team will keep the second. All consent forms will be in paper format. Signed consent forms will be submitted to team supervisors at the end of each day of data collection. Participants will not receive financial incentives as part of the recruitment process. Instead, a Mpox flyer with key prevention and safety messages will be provided to reinforce protective behavior. Data collectors will verbally explain the information on the flyer if the participant is illiterate.

# 4.0 Administrative Considerations

## 4.1 Data Management Systems

All data collection activities will be done on-site or off-site (depending on internet availability) using a web-based REDCap system to store all project data. The REDCap system will ensure that the country-specific databases are uploaded from the member state. Africa CDC will host the REDcap data server with each member state having a replica copy of the database. Access to the data will be strictly protected, restricted, and only available through official request and site-specific access will be granted to each member state team to view and edit their respective data.

# 5.0 Appendices

5.1 Mpox Risk Categorization

| **No.** | **Description** | **Countries as of 27 August 2024** |
| --- | --- | --- |
| 1 | Countries experiencing sustained human-to-human transmission | DRC, Burundi, Nigeria, South Africa, |
|  |  | Cote d’Ivoire, Central African Republic |
|  |  |  |
| 2 | Countries not already falling into Category 1 but experiencing sporadic human cases since 01 January 2022 | Rwanda, Kenya, Uganda, Sierra |
|  |  | Leone, Liberia, Ghana, Cameroon, |
|  | and / or, countries that are assessed as having endemic zoonotic reservoirs for mpox | Gabon, Republic of Congo, Morocco, |
|  |  | Egypt, Benin, Mozambique, Sudan |
| 3 | Countries not already falling into Category 1 or Category | Angola, Zambia, Eswatini, Lesotho, |
|  | 2 that are assessed as requiring enhanced readiness | Ethiopia, South Sudan, Tanzania, |
|  | including due to proximity to Category 1 countries by land air or sea | Malawi, Republic of Guinea |
|  |  |  |
| 4 | All other countries |  |

# 5.2 Schedule

| **Date in month** | **Activity** | **Responsible** | **Deliverable** | **Remarks** |
| --- | --- | --- | --- | --- |
| August 2024 | Develop the proposal | Mary | Proposal developed |  |
| Sept 2024 (first week) | Proposal undergoes review by Ethicists and in country scientist | Dr Fallah | Proposal reviewed and ethics approval secured |  |
| Sept 2024 (third week) | Data tools translated into French, Swahili and Lingala | National Coordinators for Phase 1 selected AU MS | Tools translated and pretested |  |
| Sept 2024 (Third week) | Data collection team trained | Mary | Trained team of data collection |  |
| Sept -Oct 2024 | Data collection  And ongoing analysis | STO Western, Eastern and Central RCCs supported by RCCE leads | Data collected and analyzed |  |
| October 2024 | Application of findings | Member State team led by Dr Fallah and Africa CDC team led by Mary | RCCE plans developed informed by such evidence |  |

# 5.3 Budget

# 5.4 Informed Consent Form

Introduction:

Welcome to this session, we appreciate your willingness to complete this brief survey. This survey aims to understand better access and attitudes to Mpox disease and Mpox vaccination among communities as well as healthcare workers. You are being asked to participate in this survey because your views are important in the control of Mpox disease. The survey will last about 30 to 45 minutes.

The risks of being involved with this survey are minimal. There are no penalties for not participating in this survey. For example, you will have no employment repercussions if you choose not to participate. Participation is entirely voluntary, and you may decide to discontinue your participation at any point. You have no direct costs or benefits for participating in this survey. However, your participation could help us understand how to best target interventions and other preventive measures to keep communities safe during this outbreak.

Your participation in this survey will be strictly confidential. There are no wrong or right answers and all information given will be considered important to us. None of the information you share with us will be associated with your name. Only staff involved in the analysis and/or review of the survey responses will access the data. Every effort possible will be taken to ensure your privacy, including data de-identification. There may be unforeseeable risks hence we can’t guarantee 100% privacy. You can end your participation without penalty at any point during this survey. Thank you for your time.

May we proceed with the survey?

□ Yes, we can proceed

□ No, I do not want to proceed (STOP and identify another participant and complete a new questionnaire form)

# 6.0 References

1.	Jafari K, Woodward GA. Mpox. Pediatr Emerg Care. 2023;39(11):883-9.

2.	Okwor T, Mbala PK, Evans DH, Kindrachuk J. A contemporary review of clade-specific virological differences in monkeypox viruses. Clin Microbiol Infect. 2023;29(12):1502-7.

3.	Van Dijck C, Hoff NA, Mbala-Kingebeni P, Low N, Cevik M, Rimoin AW, et al. Emergence of mpox in the post-smallpox era-a narrative review on mpox epidemiology. Clin Microbiol Infect. 2023;29(12):1487-92.

4.	Ramírez-Soto MC, Arroyo-Hernández H. Monkeypox virus infections in low-risk groups during the 2022-23 global outbreak: An analysis of the WHO global report. Infez Med. 2024;32(1):12-9.

5.	Ndembi N, Folayan MO, Ngongo N, Ntoumi F, Ogoina D, El Rabbat M, et al. Mpox outbreaks in Africa constitute a public health emergency of continental security. Lancet Glob Health. 2024.

6.	Carneiro FRO, Daxbacher ELR, DaCosta A, de Sá Gonçalves H, Ramos ESM, Penna GO. Monkeypox: What the Dermatologist Needs to Know. Skinmed. 2024;22(1):18-27.

7.	Brown K, Leggat PA. Human Monkeypox: Current State of Knowledge and Implications for the Future. Trop Med Infect Dis. 2016;1(1).

8.	Mitjà O, Alemany A, Marks M, Lezama Mora JI, Rodríguez-Aldama JC, Torres Silva MS, et al. Mpox in people with advanced HIV infection: a global case series. Lancet. 2023;401(10380):939-49.

9.	Thornhill JP, Gandhi M, Orkin C. Mpox: The Reemergence of an Old Disease and Inequities. Annu Rev Med. 2024;75:159-75.

10.	Zucker J, Hazra A, Titanji BK. Mpox and HIV-Collision of Two Diseases. Curr HIV/AIDS Rep. 2023;20(6):440-50.

11.	Jezek Z, Khodakevich LN, Szczeniowski MV. [Human monkey pox: its clinico-epidemiological characteristics]. Zh Mikrobiol Epidemiol Immunobiol. 1988(6):23-30.

12.	Ogoina D. COVID-19: The Need for Rational Use of Face Masks in Nigeria. Am J Trop Med Hyg. 2020;103(1):33-4.

13.	Ogoina D, Yinka-Ogunleye A. Sexual history of human monkeypox patients seen at a tertiary hospital in Bayelsa, Nigeria. Int J STD AIDS. 2022;33(10):928-32.

14.	Mbala PK, Huggins JW, Riu-Rovira T, Ahuka SM, Mulembakani P, Rimoin AW, et al. Maternal and Fetal Outcomes Among Pregnant Women With Human Monkeypox Infection in the Democratic Republic of Congo. J Infect Dis. 2017;216(7):824-8.

15.	Parkhurst JO. Understanding the correlations between wealth, poverty and human immunodeficiency virus infection in African countries. Bull World Health Organ. 2010;88(7):519-26.

16.	Cao Z, Liu T, Li X, Wang J, Lin H, Chen L, et al. Individual and Interactive Effects of Socio-Ecological Factors on Dengue Fever at Fine Spatial Scale: A Geographical Detector-Based Analysis. Int J Environ Res Public Health. 2017;14(7).

17.	Sahasranaman A, Jensen HJ. Poverty in the time of epidemic: A modelling perspective. PLoS One. 2020;15(11):e0242042.

18.	Tambo E, Al-Nazawi AM. Combating the global spread of poverty-related Monkeypox outbreaks and beyond. Infect Dis Poverty. 2022;11(1):80.

19. Awoyomi OJ, Njoga EO, Jaja IF, Oyeleye FA, Awoyomi PO, Ibrahim MA, Saulawa MA, Galadima HB, Rowaiye AB, Olasoju TI, Idrisa JA, Olalere FDH, Olasoju MI, Adisa OH, Adetunji VE, Idemudia OO, Ezenduka EV, Oguttu JW. Mpox in Nigeria: Perceptions and knowledge of the disease among critical stakeholders-Global public health consequences. PLoS One. 2023 Mar 30;18(3):e0283571. doi: 10.1371/journal.pone.0283571. PMID: 36996122; PMCID: PMC10062623.

## Qualitative Checklist Tool

**SOCIO-ECOLOGICAL BEHAVIORAL DRIVERS OF MPOX DISEASE IN SELECTED AU MS FOR DIFFERENT THEMES**

| ***Study area profile*** | | |
| --- | --- | --- |
| Ecosystem descriptor |  | |
| Administrative Area |  | |
| Human settlement type and name |  | |
| Peridomestic animal community farmers |  | |
| Main economy process |  | |
| Agriculture production system |  | |
| Affected community |  | |
| Housing Standard |  | |
| Infrastructure |  | |
| Health system |  | |
| Water resources |  | |
| Energy source |  | |
| Environmental state and pressure |  | |
| Vector reservoir |  | |
| Political structure |  | |
| Actors influencing disease event |  | |
| ***Exploring external shocks*** | | |
| Climate |  | |
| Conflict |  | |
| Economic |  | |
| ***Plausible Health Policies*** | | |
| Education on avoidance |  | |
| Nutrition |  | |
| Prevention and control intervention |  | |
| Control measures |  | |
| Treatments |  | |
| ***Socio demographic factors*** | | |
| Age | |  |
| Sex | |  |
| Occupation | |  |
| Infected | |  |
| Affected | |  |
| Housing type | |  |
| Marital status | |  |
| Education status | |  |
| Religious status | |  |
| **Stigma and Discrimination** | |  |
| Feeling of Isolation | |  |
| Stereotype | |  |
| Blame | |  |
| Anxiety and Fear | |  |
| Depression and suicide | |  |
| **Vaccine perceptions** | |  |
| Knowledge about vaccines | |  |
| Sources of trusted information | |  |
| Self-efficacy | |  |
| Support system | |  |
| Availability of vaccines | |  |
