# Supplementary material for: Community knowledge and perceptions of social, ecological, and behavioural risk factors for mpox in Burundi: A nationwide cross-sectional survey
Source: PLOS Glob Public Health. 2026 Oct 27;6(10):e0007383. doi: 10.1371/journal.pgph.0007383 (PMC13712512; doi:10.1371/journal.pgph.0007383)
Supplement: Supplementary file 2 [file pgph.0007383.s002.docx]

**Field implementation procedures for community-based household surveys**

**1. Preparation of the field team**

- Before going to the field, the data collectors and supervisors receive training on the study, the sampling procedure, household selection, participant eligibility, consent, questionnaire administration, confidentiality, and use of the data collection tool.
- Each team has a supervisor who follows the activities of the team and provides support during data collection.

**2. Arrival in the community**

- The team goes to the selected community according to the sampling plan.
- The team introduces the study to the local authorities or community leaders when necessary and explains the purpose of the visit.
- The team works in the communities selected for the study and follows the sampling plan provided by the study coordination team.

**3. Selection of households**

- Households are selected according to the procedure defined in the study protocol.
- Depending on the study, households may be identified through household listing, mapping, systematic selection, random selection, or another method specified in the sampling plan.
- The same procedure is followed in all selected communities.
- Households are not selected simply because they are easier to reach or closer to the field team.

**4. Contact with the household**

- When the team arrives at a selected household, the data collector introduces the study and explains the purpose of the visit.
- The data collector checks whether the household and potential participants meet the study criteria.
- The study information is provided in a language understood by the participant, and questions are answered before the interview starts.

**5. Selection of participants and consent**

- Participants who meet the eligibility criteria are invited to participate.
- Participation is voluntary. The data collector explains the study, the interview, confidentiality, and the participant's right to refuse or stop the interview.
- Consent is obtained before starting the interview, following the requirements of the study protocol.
- Where applicable, the required consent from a parent or guardian and assent from the participant are obtained.

**6. Data collection**

- The questionnaire approved for the study is administered by the trained data collector.
- Questions are asked as they appear in the questionnaire. Data collectors do not suggest answers or influence the responses of participants.
- Before leaving the household, the data collector checks the questionnaire to make sure that the required information has been recorded.

**7. Household not available or refusal**

- When nobody is available at a selected household, the team follows the procedure defined in the study protocol.
- When an eligible person refuses to participate, the refusal is respected.
- A household is replaced only when this is allowed by the sampling plan and according to the procedure established for the study.

**8. Supervision**

- The supervisor regularly checks the work of the data collectors.
- This includes checking household selection, reviewing completed questionnaires, checking for missing information, and discussing problems encountered in the field.
- Any important problem or deviation is reported to the study coordinator.

**9. Confidentiality**

- Information collected from participants is kept confidential.
- Data collectors and supervisors do not share information about participants with persons who are not part of the study team.
- Paper forms, where used, are kept in a secure place. Electronic data are submitted and stored using the procedures established for the study.

**10. Field problems**

- Data collectors inform the supervisor when they encounter problems in the field.
- These may include difficulty reaching a selected community, difficulty identifying a household, repeated absence of household members, refusal to participate, problems with the electronic data collection system, or other logistical difficulties.
- The supervisor reports important issues to the study coordinator.

**11. End of field activities**

- At the end of fieldwork, the supervisor checks that the assigned communities and households have been covered according to the study plan.
- The supervisor also checks that the collected data have been submitted and that any outstanding problems have been reported.

**12. Application to specific studies**

- These procedures are used as a common guide for community-based household surveys conducted by INSP.
- Additional instructions may be provided for a specific study depending on the sampling method, study population, eligibility criteria, consent requirements, questionnaire, and organization of field activities.
